# Interaction with the future self in virtual reality reduces self-defeating behavior in a sample of convicted offenders

**DOI:** 10.1038/s41598-022-06305-5

**Published:** 2022-02-10

**Authors:** Jean-Louis van Gelder, Liza J. M. Cornet, Natascha P. Zwalua, Esther C. A. Mertens, Job van der Schalk

**Affiliations:** 1 Department of Criminology, Max Planck Institute for the Study of Crime, Security and Law, Freiburg Im Breisgau, Germany; 2 Institute of Education and Child Studies, Leiden University, Leiden, The Netherlands

**Keywords:** Risk factors, Psychology, Human behaviour, Social behaviour

## Abstract

In this study, we test an intervention in which convicted offenders interacted with an age-progressed avatar representing their future selves in virtual reality. During the interaction, they reflected on their current lifestyle, alternating between the perspective of their present self and that of their future self. We hypothesized that this embodied experience would increase their ability to imagine themselves in the future and reduce their engagement in self-defeating behavior, as measured with a self-report survey. In line with expectations, results indicated that the interaction increased vividness of the future self compared to baseline and reduced self-defeating behavior, including alcohol use and overspending, one week later. In addition, increases in vividness were associated with a reduction in self-defeating behavior over and above other concepts relating to the future self, including connectedness, similarity, and valence. The results are based on a small sample and should therefore be considered as indicative of the possibilities of our virtual reality paradigm as an intervention tool to reduce self-defeating behavior.

## Introduction

Our future and the realization that we will grow older are not always on the forefront of our minds. There is ample research showing that many people excessively discount the future and make decisions that run counter to their long-term self-interests^1^. One group for which the inclination towards short-term gratification is particularly pronounced is criminal offenders. Compared with their normative peers, offenders are more likely to be substance abusers, to overspend and save too little, and more often engage in reckless activities, such as speeding, driving under influence, and unprotected sex^2–5^. Similar to delinquency, these behaviors are characterized by immediate benefits and more delayed costs. Offenders, in other words, tend to be short-sighted decision makers across a series of correlated self-defeating domains. Although this group stands to benefit from interventions aiming to improve their ability to make informed cost–benefit tradeoffs, it has been found to be comparatively resistant to efforts to achieve this^6,7^.

The goal of this study was to stimulate future-oriented decision making in young adult offenders by drawing from theoretical perspectives that examine intertemporal decision making through the lens of present versus future selves^8–10^. Future-self models frame intertemporal choice in terms of a conflict between the needs and wants of the self at different points in time, where immediate rewards accrue to the present self and longer-term costs come at the expense of a temporally remote future self^11,12^. Psychological absence of the future self at the time of decision implies a reduced likelihood that future costs are adequately factored into the choice process, resulting in short-sighted decision making. Conversely, having vivid image of their future self may steer people away from decisions that negatively impact it, in the awareness that one day they will be that self. Rendering the future self more vivid will motivate them to act in ways that will benefit, or at least not harm, themselves in the future^13^.

Previous studies have related the ease and detail with which people are able to imagine and describe their self in the future, referred to as vividness of the future self, to improved intertemporal decision making^10,11^. Hershfield and colleagues^14^, for example, showed that individuals who interacted with a realistic version of their future self in virtual reality (VR) exhibited an increased tendency to accept delayed monetary rewards over immediate ones compared to individuals who interacted with a version of their present self. In another study using VR, university students were exposed to an age-progressed avatar representing themselves when looking into a virtual mirror^15^. Following this experience, students exposed to their future self were less likely to cheat compared to those in a control group who had seen their present self reflected in the virtual mirror. In addition, Van Gelder and colleagues^12^ found that daily interaction with a future version of the self via a social media platform led to reduced self-reported delinquent behavior among high-school students. Importantly, this reduction was mediated by corresponding changes in vividness of the future self.

In the present study, convicted offenders interacted with and virtually embodied realistic and dynamic age-progressed renderings of their future self in VR. Virtual embodiment refers to the substitution of an individual’s physical body by a virtual one, with the objective of generating the cognitive illusion that the virtual body is, at least temporarily, one’s own^16–18^. Previous research has investigated the effects of virtual embodiment on behavioral and cognitive processes^19–23^. For example, a recent study investigated the impact of virtual embodiment among male perpetrators of domestic violence^24^. Results show that after embodying an avatar representing a female victim of domestic violence, the ability to recognize fearful female faces was improved among these offenders. In another study, participants enrolled in a ‘self-counseling’ task by alternating, i.e., ‘body swapping’, between a virtual representation of themselves and an avatar resembling Sigmund Freud^23,25^. Results of this study indicated that this self-counseling task improved participants’ mood and influenced their thinking.

The current study employs a body swapping paradigm involving virtual representations of participants’ present self and future self. Convicted offenders alternated between virtually embodying their present self and their 10-year-older, ‘future’ self over multiple rounds. First, participants embodied their present self and were presented with several statements related to positive (e.g., regular exercise) and negative (e.g., drug use) behaviors. Participants were asked to indicate whether these statements applied to them or not. Subsequently, they embodied the avatar representing their future self to reflect on whether the selected behaviors were beneficial or harmful for their future self by sorting these into different stacks. After returning to embodying the present self, participants received a ‘future-self score’, based on the number of positive and negative behaviors in each stack. The exercise ended with the participant embodying the future self and providing free-format advice, which was played back to the present self.

The graphic and immersive nature of VR technology lends itself particularly well to increasing vividness of the future self. For this reason, and in line with previous research focusing on delinquent and unethical behavior^12,15^, the concept of vividness of the future self is central in this study. In addition, and extending previous work, we also include other theorized components of the psychological connection between the present self and the future self, i.e., connection to the future self, similarity to the future self, and future-self valence, and assess the extent to which each of these components is also affected by the VR task^11,26^.

Participants reported their self-defeating behavior prior to and one week after the experimental session. Vividness of the future self and the other components were measured just before, immediately following, and one week after the experimental session. In line with previous research, we hypothesized that the VR exercise would increase vividness of the future self (H1) and have a dampening effect on self-defeating behavior (H2)^12,15^. Furthermore, and also in line with earlier work, we hypothesized that decreases in self-defeating behavior would be associated with increases in vividness (H3). Aside from testing the hypotheses, we also conducted a series of exploratory analyses. First, we explored to what extent vividness was associated with self-defeating behavior over and above the other future-self concepts, i.e., connectedness, similarity, and valence. In addition, it has been argued that experiencing body ownership of a virtual avatar increases feelings of ‘presence’, the subjective sense of being in the virtual environment, which in turn increases the likelihood that the user responds realistically to virtual events^27^. Hence, we explored the relation between participants’ subjective VR experience (e.g., body ownership, presence, engagement, feelings of cognitive embodiment) and changes in vividness. Lastly, we explored whether the interaction with a vivid version of the future self affected participants’ perception of their perceived future prospects. Null hypothesis testing was conducted with *p* < 0.05 as cut-off for significant effects. We report findings where *p* < 0.10 as non-significant trends.

## Results

Means and standard deviations for all measures are listed in Table 1. There were 24 participants at T1 and T2, and 21 participants at T3, with no further missing values on any of the measures of interest. To test the hypothesis (H1) that the VR exercise was successful in increasing levels of vividness, we employed a repeated-measures ANOVA with time as a within-subjects factor. The effect of time revealed a non-significant trend [*F*(2,40) = 2.54, *p* = 0.09, η_p_^2^ = 0.11], with vividness significantly increasing from T1 to T2 [Δ = 0.492, SE = 0.196, *p* = 0.01, one-tailed^28^, *d* = 0.54] and remaining stable between T2 and T3 [Δ = − 0.111, SE = 0.212, *p* = 0.61, *d* = 0.07]. No significant changes over time were observed for any of the other future-self concepts.

**Table 1:** Means and SDs of future-self concepts and self-defeating behavior.

Measure	T1		T2		T3		η_p_^2^
	Mean	(SD)	Mean	(SD)	Mean	(SD)	
**Future self concepts**							
Vividness	4.1^a^	(1.6)	4.6^b^	(1.6)	4.5^b^	(1.4)	0.11
Connectedness	3.5^a^	(2.2)	3.8^a^	(1.9)	3.2^a^	(1.8)	0.05
Similarity	3.7^a^	(1.8)	3.8^a^	(2.0)	3.1^a^	(1.8)	0.05
Valence	4.1^a^	(0.8)	4.1^a^	(0.9)	4.2^a^	(0.8)	0.02
**Self-defeating behavior**	3.3^†^	(1.7)			2.8^†^	(2.2)	0.11
**Future time perspective**	5.2^a,b^	(0.7)	5.3^a^	(0.8)	4.9^b^	(1.0)	0.22

In total, 24 participants completed T1 and T2; 21 participants completed T3.

Means within the same row that do not share the same superscript are significantly different at *p* < 0.05.

^†^Denotes a non-significant trend at *p* < 0.10.

Next, we examined the hypothesis (H2) that self-defeating behaviors decreased as a result of the VR exercise. Means and standard deviations are presented in Table 1. There was a non-significant trend whereby self-defeating behavior decreased from T1 to T3 [*t*(20) = − 1.57, *p* = 0.07, one-tailed, *d* = 0.34]. Post-hoc exact binomial tests revealed that scores on the following items significantly decreased from T1 to T3: the proportion of participants that reported “spending more money on something than intended” was significantly lower on T3 (52.4%) than at T1 (71.4%) [*z* = − 1.69, *p* = 0.05] and the proportion of participants that reported “drinking alcohol” was significantly lower at T3 (28.6%) than at T1 (52.4%) [*z* = − 1.97, *p* = 0.02]. Scores on the remaining items did not significantly change between T1 and T3 (z scores ranged from − 1.09 to 1.12).

We subsequently tested the hypothesis (H3) that the reduction in self-defeating behaviors is associated with increases in vividness and explored to what extent the effect of vividness would persist over and above other future-self concepts, i.e., connectedness, similarity, and valence. A linear regression analysis was performed with change in self-defeating behaviors as the dependent variable, and the difference (T1-T2) scores of vividness, connectedness, similarity, and valence as predictors. Collinearity statistics indicated that multicollinearity was not a concern (see Table 2). Results showed that the overall regression model was significant [*F*(4, 16) = 3.167, *p* = 0.04], with an adjusted *R*^2^ of 0.30.

**Table 2:** Regression model predicting change in self-defeating behavior from T1 to T3.

	B	SE B	*β*	*p*	95% CI		Collinearity	
					LL	UL	Tolerance	VIF
Constant	− 0.226	0.386						
Δ Vividness	− 1.168	0.395	− 0.582	0.009	− 2.005	− 0.331	0.901	1.11
Δ Connectedness	0.138	0.179	0.148	0.452	− 0.241	0.516	0.948	1.054
Δ Similarity	0.033	0.176	0.036	0.852	− 0.339	0.406	0.961	1.041
Δ Valence	2.517	0.942	0.537	0.017	0.520	4.514	0.865	1.156

*CI* confidence interval, *LL* lower limit, *UL* upper limit.

In line with expectations, increases in vividness was significantly associated with a decrease in self-defeating behaviors (*β* = − 0.582, *p* = 0.01). Change in valence from T1 to T2 was also predictive of change in self-defeating behaviors (*β* = *0.5*37, *p* = 0.02). Because valence scores did not significantly change over time (see Table 1), we further explored this latter finding. Scatterplot inspection revealed that the positive association between change in valence and change in self-defeating behaviors was driven by the scores of a single participant. A similar scatterplot inspection of the positive association between change in vividness and change in self-defeating behavior revealed that the datapoints were more spread out and that there was a clearer linear trend in the data (footnote: Non-parametric analyses using Wilcoxon signed ranks testing revealed that the change over time in vividness [*z* = − 2.70, *p* = 0.004 (one-tailed)] was significant and the change over time in self-defeating behavior [*z* = − 1.62, *p* = 0.052 (one-tailed)] was marginally significant, suggesting that these findings were not the result of extreme values.). The scatterplots depicting the relation between change in valence and change in self-defeating behavior and the relation between change in vividness and change in self-defeating behavior can be found in the supplemental materials (Figs. S1, S2).

**Figure 1:**
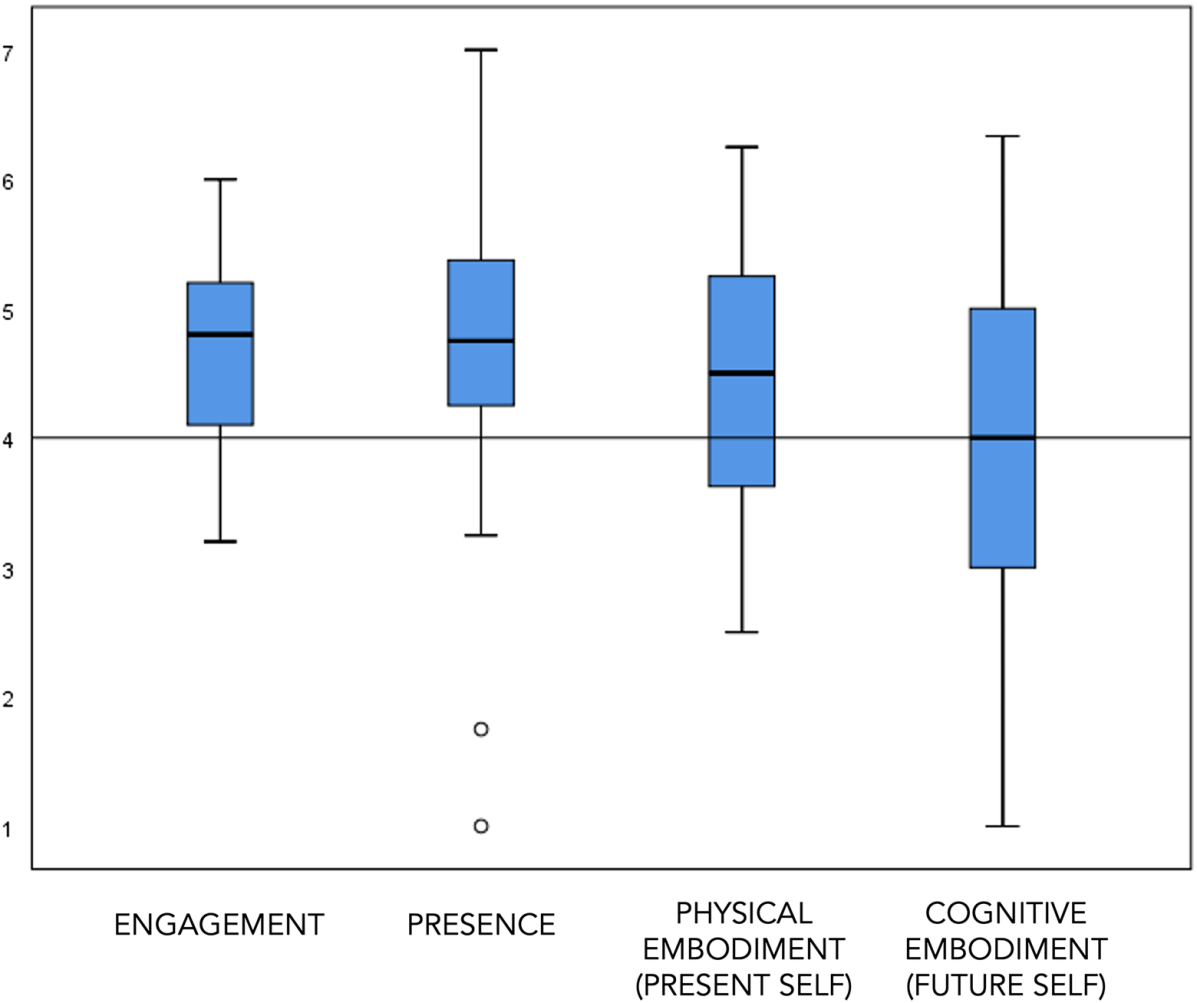
Box plots of questionnaire scores on the four VR experience measures (engagement, presence, physical embodiment of the present self, and cognitive embodiment of the future self). The value of 4 represents the mid-point of the scale (e.g., neither agree, nor disagree).

To gain further insight into the mechanisms underlying the increase in vividness from T1 to T2, we analyzed participants’ subjective VR experience (measured at T2) in terms of feelings of engagement, presence, physical and cognitive embodiment, and general discomfort, and its relationship with vividness. Figure 1 shows the means and spread of the scores on the VR experience measures. All median scores were 4 or higher (Engagement: *M* = 4.6, SD = 0.7, Median = 4.8; Presence: *M* = 4.7, SD = 1.3, Median = 4.8; Physical Embodiment: *M* = 4.4, SD = 1.1, Median = 4.5; Cognitive Embodiment: *M* = 4.0, SD = 1.3, Median = 4.0). Participants reported no (71%) or very mild levels of (29%) discomfort during the VR experience. Correlational analyses revealed no significant relations between participants’ subjective VR experience and change in vividness (all *r* < 0.20, *p* > 0.30).

Lastly, we explored whether interaction with a vivid version of the future self affected participants’ perception of their future prospects. Means and standard deviations for Future Time Perspective (FTP) are presented in Table 1. A repeated-measure ANOVA showed a significant effect of time [*F*(1.29, 25.76) = 5.78, *p* < 0.02, η_p_^2^ = 0.22] (footnote: The assumption of sphericity was violated: χ^(2)^ = 0.45, *p* < 0.001, and therefore degrees of freedom were corrected with Greenhouse–Geisser.), where the total scale score remained stable between T1 and T2 [Δ = 0.100, SE = 0.080, *p* = 0.23, *d* = 0.33], but significantly decreased post-experiment from T2 to T3 [Δ = − 0.486, SE = 0.165, *p* < 0.01, *d* = 0.50], indicating that, at T3, participants perceived reduced opportunities in life compared to T2. Additional analyses revealed that changes in FTP from T2 to T3 were unrelated to changes in self-defeating behavior (*r* = − 0.206, *p* = 0.37) and unrelated to changes in vividness from T1 to T2 (*r* = 0.245, *p* = 0.29).

A possible alternative explanation for the observed findings is that these were simply due to the time that passed between baseline and follow-up measurement. To rule out this possibility, an independent study was conducted among 23 male offenders (*M*_*age*_ = 22.9, *SD*_*age*_ = 2.9). Vividness of future self did not increase between baseline (*M*_*T1*_ = 5.23, *SD*_*T1*_ = 1.11) and follow-up one week later (*M*_*T2*_ = 4.67, *SD*_*T2*_ = 1.48) [*t*(22) = 1.56, *p* = 0.13, η_p_^2^ = 0.10], and self-defeating behavior did not significantly decrease between first (*M*_*T1*_ = 2.26, *SD*_*T1*_ = 1.51) and second measurement (*M*_*T2*_ = 2.13, *SD*_*T2*_ = 1.60) [*t*(22) = 0.47, *p* = 0.64, η_p_^2^ = 0.01]. Furthermore, no significant differences were observed for any of the other future self concepts (connectedness, similarity, and valence) or future time perspective, all *t*s < 1.31, all *p*s > 0.20, all η_p_^2^ < 0.07 (see supplemental materials S1 for details).

## Discussion

We theorized that ‘bringing the future self to life’ through VR would help individuals to realize that they will be this future self one day and to motivate them to take the longer-term consequences of their choices into account. In order to generate dynamic renderings of the future self, we capitalized on VR technology to not only expose participants to their age-progressed self but also to embody it. Participants engaged in an exercise designed to make them reflect on their current lifestyle as if they were looking back on their lives. Furthermore, the exercise allowed participants to both provide and listen to the advice of their older self.

This study’s main finding is that offenders’ interaction with an avatar representing their future self decreases engagement in self-defeating behavior through increases in vividness of the future self. Importantly, vividness of the future self was found to be associated with reduced self-defeating behavior over and above other relevant concepts related to the future self, i.e., connectedness, similarity, and valence. While connectedness, similarity, and valence have been related to positive future-oriented behavior in previous studies, e.g., reduced delay discounting^29^, improved academic performance^30^, increased exercise, and better mental health and well-being^31^, our findings suggest that (lack of) vividness of the future self is the only concept impacting on self-defeating behavior. Although valence of the future self was also related to self-defeating behavior—in such a way that participants whose image of their future self became more positive also reported increases in self-defeating behavior—average valence scores did not significantly change between the different measurement points.

It should be noted that valence was measured on a five-point scale and with a single item. It is possible that the measure gave too little scope for participants to demonstrate a change in the perception of their future self in terms of valence. However, although the mean of valence at first measurement was above the midpoint of the scale, it was not at the top end of the scale, making it unlikely that the lack of effect was due to a ceiling effect. Future research could further investigate how concepts that describe the perception of the future self can be measured in a comprehensive and meaningful way, including (but not limited to) valence, connectedness, and similarity.

Exploratory analyses revealed no relationship between how participants experienced the VR manipulation and changes in vividness scores. Closer inspection revealed high individual variation on the subjective VR experience measures whereas the corresponding mean and median scores appeared to be slightly lower than previous VR manipulation studies^22,23,32^. Further research is needed to verify which VR experience contributors underly cognitive and behavioral changes through VR.

To examine the possibility that other factors than the VR exercise could have influenced vividness scores and subsequent behavior, we conducted a separate study as a post-hoc examination without the experimental manipulation, in which we measured the future self constructs, including vividness, and self-defeating behavior at two different time points. The results of this study showed no significant changes over time for any of the future self variables nor for self-defeating behavior. Aside from lending further support to the hypotheses, these findings suggest vividness is a relatively stable construct, at least over time-scales similar to the one of the present study.

Furthermore, results indicated that, after having interacted with their future selves, participants reported feeling more limited in their future prospects one week later. This appears at odds with research suggesting that intertemporal decision making is improved when individuals are reminded of their future^14^. However, it is important to note that the current study was conducted with a sample of convicted offenders. It is plausible that the VR exercise made them aware that their opportunities in life had become more restricted due to their previous choices. Possibly, this confrontation with the limiting effect of their own past choices is a necessary precondition for making positive life changes. Further investigation is required to understand how exposure to the future self affects one’s perception of future time and how this is related to daily decision-making processes.

The current study presents the first findings of a novel intervention currently being developed to reduce self-defeating behavior among offenders. The results provide preliminary evidence that increasing the vividness of the future self reduces self-defeating behavior and contributes to more future-oriented decision making among this group. We acknowledge that the findings are modest and thus only provide a first tentative indication of the usability of VR as a tool to increase future oriented behavior in a population of criminal offenders. Further research is required to improve our understanding of what sort of changes in future self conception would impact behavior changes on longer timescales, e.g., months or years out.

Despite the small sample size and modest behavioral effects, we believe the results hint at the potential of the future self VR paradigm and observe that even small decreases in crime rates can have large effects in terms of societal and social costs^33^. We suggest that the intervention can serve as a template for offender treatment programs while at the same time we acknowledge that other approaches aiming to increase vividness of the future self than VR^12^ may achieve similar effects. Especially since existing correctional intervention and reentry programs vary strongly in their success rates in terms of reducing recidivism^6,34,35^, it is imperative to continue to explore novel ways to reduce delinquent and other types of self-defeating behavior.

## Methods

### Sample

A total of 24 convicted male offenders (*M*_*age*_ = 23.7, *SD*_*age*_ = 3.7) participated in the study. Recruitment and data collection took place at four locations of the Dutch Probation Service (N = 20) and in a penitentiary institution in the Netherlands (N = 4) from January 2020 to March 2020. Research was performed in accordance with relevant guidelines/regulations and ethical approval was obtained from the Ethics Committee Behavioural, Management and Social sciences, University of Twente (#191347).

### Materials

The study had three measurement points: immediately before (T1), immediately after (T2), and one week after the experimental session (T3). Participant responses were collected with Qualtrics survey software (footnote: www.qualtrics.com, version January 2020.).

#### Future-self concepts

The vividness of the future self measure was an abbreviated version of the measure proposed by Van Gelder and colleagues^12^ and consisted of three items that were measured on 7-point Likert scales (*completely disagree* to *completely agree*), e.g., “I find it easy to imagine myself in the future”. Alpha reliability was adequate for each of the three measurement points (T1, T2, T3) α = 0.81 to α = 0.92. Connectedness and similarity were each measured on a 7-point scale marked with pairs of circles that varied in their degree of overlap ranging from no overlap to almost complete overlap^36^. Participants were asked to select a pair of circles that best described how connected and similar they felt to their self ten years into the future. Valence of the future self^36^ was measured with a slider measure depicting five different emoticons that varied in positivity (*very sad* to *very happy*) (footnote: During piloting the materials of the study, it became clear some participants had difficulty distinguishing between the scale points of the 7-point scale. To reduce participant burden, the scale was therefore reduced to a 5-point scale.).

#### Self-defeating behavior

Self-defeating behavior was assessed at T1 and T3 using a self-report format. In total, nine items were used to inquire about delinquent, impulsive, and other self-defeating behaviors in the preceding seven days and were based on Van Gelder et al. (2015) but adapted to fit the current sample (footnote: Items (preceded by the stem ‘How often in the past week did you…’) were: 1) miss school or work? 2) spend more money than you intended to? 3) do or say something you regretted later? 4) drink alcohol? 5) use drugs? 6) use violence against someone? 7) make a decision without thinking? 8) damage something? 9) steal something?). Answer categories were *never, 1 or 2 times, 3 to 5 times, 6 to 10 times, more than 10 times.* Previous research has indicated higher reliability and validity and lower skewness of ‘variety scales’ compared to frequency scales^37,38^. In line with this, responses on the self-defeating behavior measure were recoded to a dichotomous score of 0 (“never”) or 1 (“more than once”) for each item and were subsequently summed to a scale score. Alpha reliability ranged from 0.55 (T1) to 0.74 (T3).

#### Future time perspective

To explore participants’ perception of their future prospects, the 10-item Future Time Perspective scale (FTP)^39^ was employed. FTP measures perceptions about what is possible in one’s remaining lifetime and the awareness of constraints and barriers of one’s future time using 7-point Likert scales (*completely disagree* to *completely agree*). Alpha reliability was acceptable for all three measurement points (T1, T2 and T3) (α ≥ 0.75).

#### VR experience

Directly after the VR task, at T2, we measured several aspects of participants’ experience of the VR task. Since the target group was not assumed to be highly familiar with survey research, we limited the burden on participants by using abbreviated versions of the following measures. To measure feelings of physical embodiment of the Present Self (PS) avatar we used 4 items from Banakou et al. (2018)^32^ (α = 0.65). To measure cognitive embodiment of the Future Self (FS) avatar, we developed a 3-item scale measuring the extent to which participants experienced a change in thinking during the embodiment of the Future Self (FS) avatar (α = 0.73), e.g., “When sitting in the chair of my future self, my thoughts were different from normal”. Presence was measured using 4 items from the Spatial Presence Experience Scale^40^ (α = 0.91), and Engagement was measuring with 5 items from the User Engagement Scale^41^, which consisted of the subscales: Focused Attention (3 items, α = 0.67) and Reward (2 items, α = 0.79). Responses for all items were measured on 7-point scales (*completely disagree* to *completely agree*). One item from the Virtual Reality Sickness Questionnaire measured feelings of discomfort during the VR experience^42^, i.e., “Did you experience a general feeling of discomfort during the VR exercise?”, measured on a 4-point scale (*not at all* to *severely)*.

### Equipment

The virtual environment was developed with the Unity Pro engine (footnote: Unity Pro engine version 2018.4.xx was used. The environment was developed by Studio Barbaar (www.studiobarbaar.com).). An MSI GS63VR 7RG-043NL gaming laptop running Windows 10 through an Intel i7 CPU, with a NVIDIA GeForce GTX 1070 MAX-Q graphics card and 16 GB of RAM was used to render the virtual environment. Furthermore, an HTC Vive head-mounted display (HMD) with a 110° field-of-view and a resolution of 1080 × 1200 pixels per eye displayed at 90 Hz was used. Two base stations allowed 360° movement. Participants used two controllers to move the arms and torso of their avatar.

Pictures of participants’ faces were taken on site. Avatars were created using plug-in software (footnote: To create digital representations of participants we used software developed by Avatar SDK, version ‘Head 1.2’ (www.avatarsdk.com). An age-progression algorithm was used to create the FS representation (footnote: Aging software developed by Change My Face (www.changemyface.com) was used to create the future self avatar.). The experimenter adjusted the avatar body to the proportions of the participant’s actual body.

### Procedure

**Figure 2:**
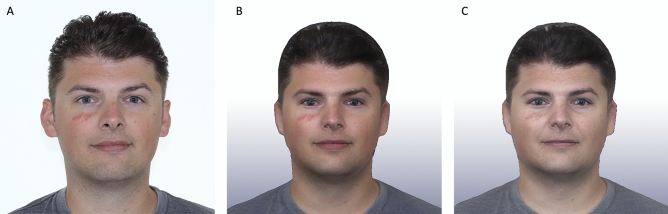
Avatar creation process. (**A**) Source image; (**B**) digital avatar version reflecting present self; (**C**) aged avatar version reflecting Future Self. Informed consent was obtained from the participant for publishing their image.

**Figure 3:**
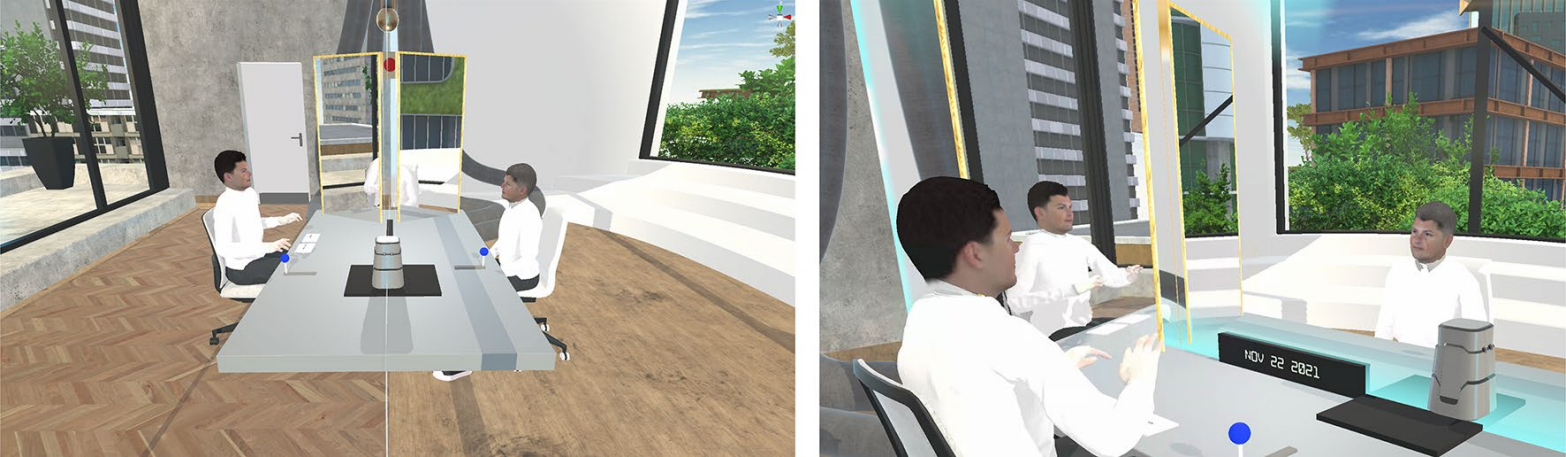
Impressions of the virtual reality environment in which the present self (PS) (left) interacts with his Future Self (FS) avatar (right). The mirrors at the far end of the table serve to foster virtual embodiment. Pushing the blue levers on either side of the table activates the ‘time machine’ to switch perspective from the PS to the FS or vice versa.

Prior to the start of the experimental session, participants provided informed consent and completed the baseline measures (T1). Meanwhile, the PS and FS avatars were created by the experimenter (see Fig. 2). Next, participants were equipped with the HMD and noise-cancelling headphones, after which they entered the virtual environment (see Fig. 3). Participants first embodied their PS avatar. Once immersed in VR, a virtual robot controlled by the experimenter guided participants through the experiment (the experimenter’s voice was distorted to sound like a robot). To foster acceptance of the PS avatar, participants were asked to perform a series of physical movements in front of the virtual mirror resulting in real-time movement synchrony of the virtual body. This movement synchrony was established by inverse kinematics based on tracking information from the HMD and handheld controllers^43^.

Next, participants were requested to sort a virtual stack of 24 cards, each displaying a positive or a negative statement related to health, financial situation, or social contacts (e.g., “I take care of my health”, “I have debts”, “I avoid people who are bad for me”) by placing them into either a YES or a NO box, depending on whether the statement applied to them or not. After completing this task, participants ‘time-travelled’ to the other side of the virtual table by pulling a (virtual) lever located on the table, to embody their FS avatar. Participants were instructed to look into the mirror to their right and saw their aged avatar reflected in it. Subsequently, participants again performed several movement exercises while looking into the mirror to foster FS embodiment. Participants were then asked to reflect on the cards that they had put in the YES box earlier and sorted these into either a GOOD FOR ME box or a BAD FOR ME box. Once completed, participants time-travelled back to their PS avatar where they saw their ‘future-self score’ reflecting the total number of cards that had been sorted into the GOOD FOR ME and BAD FOR ME boxes by the FS avatar. Next, participants were asked several scripted questions (e.g., “Does your future self benefit from your current lifestyle?”). After a few minutes of reflection, participants time-travelled to embody their FS again to come up with some advice for their PS. This advice was recorded and, once participants switched to their PS for the last time, played back to them.

After the VR exercise, participants completed the T2 measures. One week after the VR experiment, participants received an email with a request to complete the follow-up measures (T3) (footnote: Detained participants (n = 4) received a paper version of the follow-up measures by an experimenter. Furthermore, in line with prison regulations, detained participants were not compensated by a €10 gift voucher.). After completing the follow-up measures, participants were debriefed and received a €10 gift voucher.

## Supplementary Information

Supplementary Information.

## Data Availability

The dataset generated and analyzed during the current study and corresponding syntax are available from the first author on reasonable request.
