# Supplementary material for: Interaction with the future self in virtual reality reduces self-defeating behavior in a sample of convicted offenders
Source: Sci Rep. 2022 Feb 10;12:2254. doi: 10.1038/s41598-022-06305-5 (PMC8831494; doi:10.1038/s41598-022-06305-5)
Supplement: Supplementary file 1 — Supplementary Information. [file 41598_2022_6305_MOESM1_ESM.docx]

**Interaction With the Future Self in Virtual Reality Reduces Self-Defeating Behavior in a Sample of Convicted Offenders**

Jean-Louis van Gelder, Liza J.M. Cornet, Natascha P. Zwalua, Esther C.A. Mertens, Job van der Schalk

**Supplemental materials**

**Method**

*Sample*. Young male offenders were recruited via the Dutch Probation Service. There were 35 participants at baseline (after exclusion of 3 participants whose responses were not recorded accurately). At follow-up, 23 participants (*M_age_* = 22.9, *SD_age_* = 2.9) completed the study (after exclusion of 2 participants who had missing values on all variables, 2 participants who had duplicate entries, and 4 participants whose data was not recorded accurately).

*Materials*. The study was administered online via Qualtrics survey software and had two measurement points: baseline (T1) and follow-up one week later (T2). The same questionnaires that were used in Study 1 were administered: Future-Self concepts (vividness of future self: α_T1_ = .65, α_T2_ = .86), Self-Defeating Behavior (α_T1_ = .42, α_T2_ = .42), and Future Time Perspective (α_T1_ = .85, α_T2_ = .89).

*Procedure*. At baseline participants provided informed consent and answered some general background questions before responding to the Future-Self Concepts questionnaire, the Future Time Perspective questionnaire, and the Self-defeating behavior questionnaire. They then had the opportunity to leave comments, were thanked and reminded about the follow-up study one week later. The link to the follow-up survey was sent to participants via email. Participants again filled out the Future-Self Concepts questionnaire, the Future Time Perspective questionnaire, and the Self-defeating behavior questionnaire. They were then thanked, debriefed, and received a voucher redeemable for €10.

**Results**

Table S1 provides an overview of the findings. In contrast to Study 1, vividness of future self did not increase between baseline and follow-up a week later (in fact, there was a small non-significant decrease), and self-defeating behavior did not significantly decrease between first and second measurement. No significant differences were observed for any of the other measurements.

|  | Baseline | | Follow-up | | *t* | *p* | η_p_^2^ | Cohen’s *d* |
| --- | --- | --- | --- | --- | --- | --- | --- | --- |
| Measure | Mean | (SD) | Mean | (SD) | (df = 22) |  |  |  |
| *Future Self Concepts* |  |  |  |  |  |  |  |  |
| Vividness | 5.23 | (1.11) | 4.67 | (1.48) | 1.56 | .13 | .10 | 0.32 |
| Connectedness | 3.91 | (1.91) | 4.04 | (1.75) | -0.28 | .78 | .004 | 0.06 |
| Similarity | 3.43 | (1.44) | 3.65 | (1.72) | -0.54 | .59 | .01 | 0.11 |
| Valence | 3.96 | (1.11) | 4.13 | (0.87) | -0.81 | .43 | .03 | 0.17 |
| *Self-defeating behavior* | 2.26 | (1.51) | 2.13 | (1.60) | 0.47 | .64 | .01 | 0.10 |
| *Future Time Perspective* | 4.57 | (1.11) | 4.37 | (1.15) | 1.31 | .20 | .07 | 0.27 |

**Table S1.** Means and SDs of future-self concepts, self-defeating behavior, and future time perspective, with *t*, *p*, η_p_^2^ and Cohen’s *d* values for the comparison between measurement points. There were no significant differences between measurement points for any of the variables.


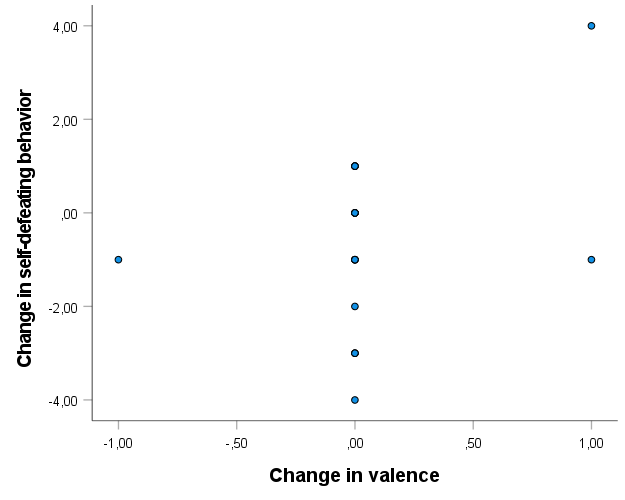


**Figure S1.** Scatterplot of the relation between change in valence and change in self-defeating behavior.


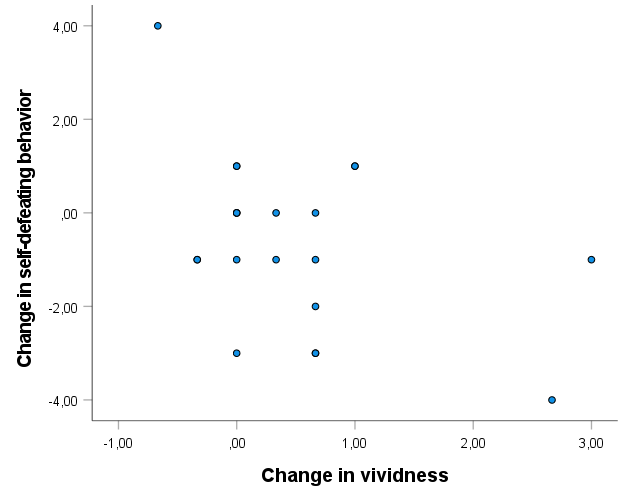


**Figure S2.** Scatterplot of the relation between change in vividness and change in self-defeating behavior.
